# Epigenetic mechanisms in Tendon Ageing

**DOI:** 10.1093/bmb/ldaa023

**Published:** 2020-08-22

**Authors:** Kiran Riasat, David Bardell, Katarzyna Goljanek-Whysall, Peter D Clegg, Mandy J Peffers

**Affiliations:** Department of Musculoskeletal Biology, Institute of Life Course and Medical Sciences, William Henry Duncan Building, 6 West Derby Street, Liverpool L7 8TX, UK; Department of Musculoskeletal Biology, Institute of Life Course and Medical Sciences, William Henry Duncan Building, 6 West Derby Street, Liverpool L7 8TX, UK; Institute of Veterinary Science, University of Liverpool, Leahurst Campus, Neston, Wirral CH64 7TE, UK; Department of Musculoskeletal Biology, Institute of Life Course and Medical Sciences, William Henry Duncan Building, 6 West Derby Street, Liverpool L7 8TX, UK; Department of Musculoskeletal Biology, Institute of Life Course and Medical Sciences, William Henry Duncan Building, 6 West Derby Street, Liverpool L7 8TX, UK; Department of Musculoskeletal Biology, Institute of Life Course and Medical Sciences, William Henry Duncan Building, 6 West Derby Street, Liverpool L7 8TX, UK

**Keywords:** tendon, ageing, epigenetics, histone modification, non-coding RNAs, DNA methylation

## Abstract

**Introduction:**

Tendon is a composite material with a well-ordered hierarchical structure exhibiting viscoelastic properties designed to transfer force. It is recognized that the incidence of tendon injury increases with age, suggesting a deterioration in homeostatic mechanisms or reparative processes. This review summarizes epigenetic mechanisms identified in ageing healthy tendon.

**Sources of data:**

We searched multiple databases to produce a systematic review on the role of epigenetic mechanisms in tendon ageing.

**Areas of agreement:**

Epigenetic mechanisms are important in predisposing ageing tendon to injury.

**Areas of controversy:**

The relative importance of epigenetic mechanisms are unknown in terms of promoting healthy ageing. It is also unknown whether these changes represent protective mechanisms to function or predispose to pathology.

**Growing point:**

Epigenetic markers in ageing tendon, which are under-researched including genome-wide chromatin accessibility, should be investigated.

**Areas timely for developing research:**

Metanalysis through integration of multiple datasets and platforms will enable a holistic understanding of the epigenome in ageing and its relevance to disease.

Tendinopathies are a significant cause of morbidity in both human and animal species, accounting for up to 50% of musculoskeletal injuries presented for medical^1^ or veterinary^2^ attention. As ageing is a key risk factor in the development of tendinopathy, it is essential to understand the mechanism that predisposes failure. This review summarizes the literature on the epigenetic mechanisms identified in ageing healthy tendon to date.

**Fig. 1:**
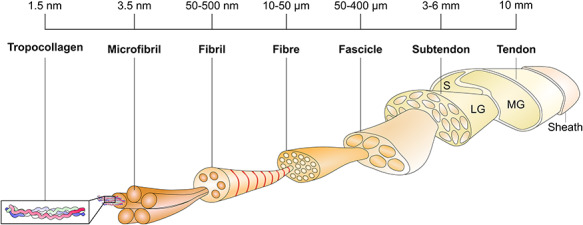
Schematic representation of the highly ordered structure of tendon tissue. This figure was made by Neil Marr, Royal Veterinary College, London 2020 specifically for this review.

The hierarchical structure of tendon has been well defined (Fig. 1)^3^, and tendons have been sub-classified into those which act to store and return energy during locomotion (energy storing), such as the Achilles tendon, and those which are involved with maintaining body position (positional). Although the basic tendon structure is similar, there are recognized differences conferring altered mechanical properties including ageing.^4^ Energy storing tendons are more prone to regular high impact, and the transfer of force from muscle to bone renders them more susceptible to micro tears ultimately leading to tendinopathy.^5^

Studies of the extracellular matrix composition of these two tendon types revealed elevated glycosaminoglycans, increased abundance of cartilage oligomeric matrix protein and a requirement for lubricin and elastin in energy storing tendon, enabling the energy storing tendon to retain its ‘spring’-like trait.^6^ However, in ageing, there is evidence for protein alterations.^7^ The molecular and cellular composition and mechanical properties of equine energy storing tendon have been shown to alter with age, due to changes in the collagenous matrix and non-collagenous matrix properties.^4^^,^^8–11^

The interfascicular matrix (IFM) also demonstrates age-related changes. This matrix compartment, comprising a complex mixture of proteoglycans, interposed between tendon fascicles, is less fatigue resistant with ageing in energy storing tendons compared to positional tendon, further supporting the notion that function and performance are significantly affected by age.^10^ Additionally, ageing is associated with an increase in IFM stiffness within energy storing tendon, reducing the elasticity of the tissue and enhancing the tendons susceptibility to micro-damage.^11^^,^^12^ Further supporting evidence of an age-related decline on the function of the energy-storing tendon comes from proteomic analysis of the IFM, suggesting that reduced protein turnover is a hallmark of ageing.^13^^,^^14^

Age-related alterations in tendon cellular function have also been identified *ex vivo,*^7^^,^^15^ with age-related changes linked to an altered tenocyte proteome and differential potential of progenitor cells to the tendon lineage. The ability of mesenchymal stem cells (MSC) to differentiate into functionally competent tenocytes also alters with age. Peffers *et al.* identified differential expression of 207 proteins between human MSCs derived from old and young donors when differentiated into tissue-engineered tendon constructs.^16^ Bioinformatics analysis identified energy and protein metabolism as the key pathways associated with age-affected proteins. Equally, equine tendon-derived differentiated tenocytes used to produce tissue-engineered constructs demonstrated distinct proteomes associated with donor ageing.^15^ A transcriptomic meta-analysis study of both tendon and tissue-engineered tendon constructs demonstrated distinct differences in how ageing affects males and females.^17^ As the incidence and anatomical location of tendinopathy is known to be influenced by sex,^18^ this difference in normal sex-related ageing may be pivotal in understanding the predisposition to, and therefore ability to prevent disease.

The ageing process affects many cellular homeostatic mechanisms^14^ such as proteostasis, gene expression regulation, response to reactive oxygen species (ROS)^19^ and, matrix remodeling, as well as a loss of regenerative capacity of tendon stem cells (TSCs). Table 1 shows emerging evidence for such age-related changes in tendon tissue. While most of these are tissue specific, many of these altered mechanisms fall in line with the hallmarks of cellular ageing.^14^

**Table 1:** Known age-related changes in tendon tissue

Characteristic	Species	Tendon type	Observed effect of age	Reference
Intrafascicular matrix	Equine	Energy storing, SDFT	Stiffness increases with age in energy storing tendon.	^80^
Collagen fibril diameter	Equine	Energy storing, SDFT	Reduces with age.	^81^
Collagen content	Equine	Energy storing SDFT	Type III collagen increased in older group.	^9^
Altered fibril arrangement	Murine	Tail tendon	Increases with age.	^82^
Glycosaminoglycans	Equine	Energy storing SDFT	Increase with age in positional tendons.	^4^
Protein turnover	Equine	Energy storing SDFT	Neopeptide number higher in young group.	^24^
Cellular senescence-inhibited gene	Rat	Energy storing, Achilles Tendon	Reduced proliferation of tenocytes. Reduced cellular senescence inhibited gene reduced in old tenocytes.	^27^
Tendon stem cells	Human	Energy storing, Achilles tendon	Pool size and functional capacity becomes exhausted with age. Reduction in both the number of TSCs, their self-renewal and differentiation potential.	^83^
Inflammageing	Equine	Energy storing, SDFT	Aged individuals exhibit a reduced capacity to resolve inflammation.	^84^
ROS	Human	Supraspinatus tendon, Rotator cuff	An increase in the expression of peroxiredoxin, a thioredoxin peroxidase with antioxidant properties suggests that oxidative stress may be involved in the pathogenesis of tendon degeneration.	^85^

SDFT; superficial digital flexor tendon, TSC; tendon stem cells, ROS; reactive oxygen species

The effect of ageing on tendon tissue has been investigated in rats,^20^ mice,^21^ horses^4^^,^^9^^,^^11^ and humans.^17^^,^^22^^,^^23^ Whilst animal models using mice and rats remain important to delineate the relationship between contributory factors to tendinopathy, these models have limitations. Rodent models lack the comparable longevity, size to mass ratio as well as the onset of the multifaceted degenerative changes known to contribute human tendon pathology. However, the parallels between human and equine tendinopathy are interesting. Both demonstrate a high prevalence that is positively associated with ageing and occupational/exercise status, with a tendency for recurrent injury.^24^^,^^25^ Additionally, structural and mechanical similarities between human and equine tendon, coupled with the longevity and athletic nature of horses, render equine tendon a useful model for investigating age and exercise-related impacts on human tendon integrity.

Currently, there is no ideal model to study the effects of the many contributory factors associated with age-related tendinopathy. Studies investigating overload and strain facilitate how some of these variables contribute to an altered phenotype but fail to address the consequence of ageing. Currently, the use of human tendon tissues in such investigations is limited as they are difficult to procure. Equally, by the time the tiossue is ready for any form of biopsy/investigation, disease is usually advanced. Healthy tissue without comorbidities is difficult to obtain making this one of the more elusive tissues to investigate thoroughly in humans.

Repair in tendinopathic tissue is closely associated with turnover of non-collagenous matrix proteins, cytokines and growth factors, without increase in production of stable long-lived collagenous matrix structures.^26^^,^^27^ The interplay between transcriptional regulation via genomic and epigenetic mechanisms may shed light on the complicated network of events that lead to appropriate tendon development and maintenance allowing a better understanding of dysregulated elements.^28^ This information could then be utilized to determine whether age-related control of expression contributes to tendinopathy.

The term ‘epigenetics’ was introduced by Waddington in 1968^29^ and is defined as the ‘interactions between genes and their products which bring phenotype into being’. Epigenetics therefore describes alterations in the regulatory mechanisms of gene expression without changes in the underlying DNA sequence.^30^ Classically considered to consist of chemical modifications to cytosine bases within DNA, and the histone packaging proteins, the discovery of microRNAs in the late 1990s and subsequent elucidation of RNA interference mechanisms added another class to this field. Thus, by regulating accessibility to, and translation of the primary genetic sequence, these processes profoundly influence cellular, and therefore tissue behavior during normal development, adaptation, and pathological processes.

Currently there is a paucity of information regarding epigenetic changes associated with the normal physiological process of ageing in tendon, as research primarily focuses on changes occurring with pathology. Many studies use injured Achilles or rotator cuff tendon models and compare to healthy tissue. Current literature aims to address age-related pathologies in a derivative way, given the known phenotypic similarities between injuried and aged tissues. Pathological tendon of any age is used as a proxy for healthy tendon, given the similarities of repetitive strain, injury and inflammatory effects on the tissue**.** No conclusive statements can be made specifically regarding ageing due to the confounding variables within these studies. Few studies investigate epigenetics alone in healthy ageing tendon tissue and the subsequent identification of the divergent mechanisms underlying age-related degeneration. Therefore, this review aims to summarize published work from the last 10 years on epigenetic changes identified in healthy ageing tendon. The implication of epigenetic mechanisms on tendon inflammation has been reviewed by Thankam *et al.,*^31^ but to the authors’ knowledge, this is the first review looking at these mechanisms in tendon ageing.

## Methods

The online databases PubMed and Google Scholar were searched using the terms ‘microRNA’ and its derivatives, ‘miR’ and ‘miRNA’; ‘long non-coding RNA’ (lncRNA); ‘small nucleolar RNA’ (snoRNA); ‘non-coding RNA’, ‘pseudogene’, ‘tendon’, ‘tendinopathy’, ‘tendinosis’, ‘ageing’, ‘DNA methylation’, ‘histone modification’ ‘ATAC-seq’ and ‘epigenetic’. Additionally, the search was restricted to the period 2009–2020.

In conjunction with the terms ‘tendon’, ‘ageing’ and ‘epigenetic’, incorporation of search terms for microRNA returned 2010 papers, ‘lncRNA’ 186 papers, ‘snoRNA’ 61 papers, and ‘pseudogene’ 125 papers. After removal of review papers, book chapters and articles not directly relevant to our terms of reference, this reduced to seven (microRNAs), four (lncRNAs), two (snoRNAs) and two (pseudogenes) papers. After accounting for papers duplicated between classes, eight articles related to non-coding RNAs remained eligible for inclusion in this review (Table 2).

**Table 2:** Non-coding RNAs identified as showing significant differential expression with age

	Gene identity	Species	Tendon type	Observed effect of age	Reference
**micro-RNAs**					
miR-1245a	Human	Achilles tendon	Reduced expression with ageing.		^23^
miR-500a-5p, miR-548j-5p, miR-618, miR-10	Human	MSCs differentiated into tenogenic tissue	miR-500, miR-548 and miR-618 increased expression with ageing. miR-10 methylation significantly increased with ageing.		^62^
26 miRs	Human	Achilles tendon	26 DE miRs identified in old versus young female-derived tissue, 4 of which (miR-1287, miR-1304, miR-1909, miR-3614) also DE in old versus young male-derived tissue. Direction of change not stated.		^17^
miR-217	Human	Achilles tendon	Tenogenic differentiation capacity of TSPCs decreases with age due to p16 induced upregulation of miR-127 resulting in reduced EGR1 expression.		^42^
miR-140-5p	Human	Achilles tendon	miR-140-5p associated with TSPC senescence via direct inhibition of Pin1 expression.		^86^
miR-135a	Rat	Achilles tendon	Down regulation of miR-135a with ageing promotes senescence in TSPCs via interaction with ROCK1.		^87^
miR-29a, miR-34a, miR-34b, miR-181b, miR-199a, miR-199b	Equine	SDFT	miRs -34b and -181b upregulated with age, miRs -29a, −34a, −199a and -199b downregulated with age.		^88^
**lncRNAs**					
45 lncRNAs of unknown function, XIST LINC00261 TSIX DLX6-AS1	Human	Achilles tendon	29 lncRNAs of unknown function increased expression with ageing. 4 functionally annotated lncRNAs overexpressed with ageing (XIST, TSIX, LINC00261, DLX6-AS1). 16 lncRNAs of unknown function reduced expression with ageing.		^23^
Not given	Human	MSCs differentiated into tenogenic tissue	5 lncRNAs identified as showing significant DE, 1 up regulated, 4 downregulated with ageing.		^7^
18 lncRNAs	Human	Achilles tendon	18 DE lncRNAs identified in old v young female-derived tissue, 2 of which (LINC00662, LINC00843) also DE in old versus young male-derived tissue. Direction of change not stated.		^17^
H19	Mouse (*in vivo*) human (*in vitro*)	Human mesenchymal and tendon-derived stem cells Murine patellar tendon	H19 accelerates tenogenic differentiation by targeting miR-29b-3p and activating TGF-β1 signaling.		^56^
**snoRNAs**					
RNVU1–6 Y-RNA	Human	Achilles tendon	RNVU1–6 increased with age (spliceosomal function). Y-RNA reduced with age (DNA replication/cell proliferation.)		^23^
SNORA1, SNORA18, SNORA25, SNORA32, SNORA40, SNORA8, SNORD5, snoU13	Human	Achilles tendon	snoU138 only DE in old versus young female-derived tissue, all others also DE in old versus young male-derived tissue. Direction of change not stated.		^17^
**Pseudogenes**					
	RP11–578024.2, AP003041.1, MKRN7P, RPS4XP22, RP11-346 M5.1, RN7SKP234, CTD-2114 J12.1, AL021068.1, MXRA5P1, RNY3P2, RP11-494 K3.2, CTC-260E6.10	Human	Achilles tendon	All functionally un-annotated; 8 upregulated and 4 downregulated with ageing.	^23^
	SDHAP2, NUTM2D, PARGP1	Human	Achilles tendon	SDHAP2, NUTM2D, PARGP1 DE in old v young female-derived tissue, PARGP1 also DE in old versus young male-derived tissue. Direction of change not stated	^17^

TSPCs, tendon stem/progenitor cells; EGR1, early growth response protein 1; Pin1, peptidyl-prolyl cis-trans isomerase NIMA-interacting 1; ROCK1, rho-associated, coiled-coil-containing protein kinase 1; TSIX, antisense transcript to XIST; LINC, long intergenic non-protein coding RNA; DLX6-AS1, *Drosophila* distal-less 6 antisense RNA 1; H19, H19 imprinted maternally expressed transcript; SNORA, small nucleolar RNA (H/ACA box); SNORD, small nucleolar RNA (C/D) box, MKRN7P, makorin ring finger protein 7; RPS4XP22, ribosomal protein S4X 22; RN7SKP234, RNA 7SK small nuclear 234; AL021068.1. ATP synthase 6; MXRA5P1, matrix remodeling associated 5 Y-linked; RNY3P2, RNA ro-associated Y3 pseudogene 2; RP11-494K3, neurofascin; SDHAP2, succinate dehydrogenase complex flavoprotein subunit A; NUTM2D, NUT family member 2D; PARGP1, poly(ADP-Ribose) glycohydrolase.

A total of 24 articles were retrieved when searching for terms related to tendon epigenetics between 2009 and 2020. Search terms included; ‘tendon’ and ‘epigenetic’, ‘DNA methylation’ and ‘tendon ageing’, ‘Tendon histone modification’, ‘Tendon ATAC-seq (Assay for Transposase-Accessible Chromatin using sequencing)’. Herein, we will discuss the regulatory properties of non-coding RNA, DNA methylation and histone modifications in relation to tendon ageing based on the literature retrieved from the past 10 years.

## Results and Discussion

### Non-coding RNAs

The non-coding RNA (ncRNA) family is conventionally subdivided into long (>200 nucleotides) and short (<30 nucleotides) non-coding subgroups.

#### MicroRNAs

These are a subclass of the small non-coding RNA (sncRNA) family and are the most extensively studied.^32^ Due to their involvement in the RNA interference (RNAi) pathway, miRNAs act as regulators of gene expression, many being highly conserved across species, indicating involvement in critical cellular processes.^33^ They are characterized by their size (21–25 nucleotides) and derivation from hairpin precursors by action of both intra-nuclear and intra-cytoplasmic RNase III enzymes. There are several pathways by which mature miRNAs can be generated, but most of the more highly conserved and abundantly expressed are believed to derive from dedicated microRNA gene loci, with about 25% being processed from introns of protein coding genes.^33^^,^^34^ The mature miRNA combines with an Argonaute protein to form the functional multi-protein RNA-induced silencing complex (RISC)^33^. Additionally, it is now understood that snoRNAs and transfer RNAs (tRNAs) can be processed by the cytoplasmic RNase III enzyme Dicer into fragments which associate with RISCs and function in a regulatory manner similar to miRNAs.^35^ MicroRNAs mediate their effects through binding principally to the 3′ untranslated region (3′ UTR) of their target messenger RNA (mRNA) with variable, but imperfect complementarity, dictated by a special ‘seed’ sequence at the 5′ terminus. The result is prevention of translation of the target into a functional protein.^36^ A significant minority of mammalian miRNAs act by directing cleavage of their mRNA target,^37^ in this respect behaving similar to plant miRNAs.

It is predicted that miRNAs influence expression of over 60% of human genes,^38^ each miRNA potentially targeting multiple mRNAs,^36^ and a single mRNA being targeted by multiple miRNAs.

Using targeted qRT-PCR analysis, Bardell *et al*.^44^ demonstrated upregulation of miRNAs -34b and -181b, and downregulation of miRNAs -29a, -34a, -199a, -199b in equine superficial digital flexor tendon (SDFT). The miR-34 family has been shown to be pro-apoptotic via suppression of sirtuin1 (SIRT1), and regulates the transforming growth factor beta (TGF-β) signaling pathway, which is essential for TSC maintenance and differentiation.^39^^,^^40^ SIRT1 is also a validated target of the miR-181 family, which has extensive regulatory functions in apoptosis and mitochondrial function, through targeting B-cell lymphoma 2 apoptosis regulator (Bcl-2) family proteins,^41^^,^^42^ ubiquitin-binding protein p62 and Parkin.^43^ miR-181 also regulates inflammation through interaction with the nuclear factor kappa-light-chain-enhancer of activated B cells (NFκB), tumor necrosis factor (TNF) and toll-like receptor 4 (TLR-4) pathways.^44^^,^^45^Down regulation of miR-29 has been associated with fibrosis in multiple organs, regulating collagen production both directly^46^ and indirectly, via the TGF-β signaling pathway^56^. The miR-199 family regulates cell survival and proliferation,^47^ targeting caveolin-2 and fibrosis^48^ Han *et al*^41^ described upregulation of miRNA-217 (a regulator of cellular proliferation and apoptosis) in rat Achilles tendon with ageing. Unbiased RNA-seq interrogation of human Achilles tendon by Peffers *et al.*^33^ identified significant downregulation of another cellular proliferation-associated miRNA, miRNA-1245a, with ageing.

As well as acting in an intracrine fashion, miRNAs also exert an endocrine-like function, being secreted into the circulation as part of a miRNA binding protein or high-density lipoprotein complex, or as part of the micro-vesicle/exosome cargo.^49^ Changes to circulating miRNAs associated with ageing and senescence have been demonstrated,^38^^,^^50–52^ suggesting age-related changes in tendon function may be an integral part of body-wide ageing processes.

#### Small nucleolar RNAs

SnoRNAs act canonically as mediators of chemical modification of ribosomal RNAs (rRNA). These 60–220 nucleotide ncRNAs primarily located within the nucleolus broadly divided into two functionally distinct categories, C/D Box and H/ACA Box, snoRNAs facilitating methylation or pseudouridylation of target RNA.^53^ Further processing of snoRNAs can generate smaller fragments displaying miRNA-like functions.^54^ RNA-sequencing analysis identified the upregulation of snoRNA RNA variant U1 small nuclear 6 (RNVU1–6) and downregulation of Y_RNA with ageing.^23^

#### Long non-coding RNAs

Characterized as ≥200 nucleotides in length, lncRNAs have recently been implicated in regulation of transcriptional processing by several methods.^55^ Proposed activity includes modification of chromatin via recruitment of histone and DNA methyl-transferases, influencing transcriptional activators and repressors, and acting as miRNA ‘sponges’, thereby removing miRNA influence on gene expression.^55^ Lu *et al*. reported that lncRNA H19 plays a key role in tenogenic differentiation by directly suppressing the action of miRNA29b-3p, promoting activity of the TGF-β1 signaling pathway.^56^ Although the authors investigated tendon healing rather than ageing, impaired capacity of stem cells to differentiate into functionally competent tenocytes with ageing has been demonstrated.^16^ The TGF-β/SMAD2/3 pathway is reportedly the most important pathway in development of limb tendons, disruption of which results in extensive loss of embryological tendon tissue. In the mature tendon.^56^ The Dysregulation of this pathway by non-coding RNAs may therefore limit the ability of the TSC pool to respond to loss of differentiated tenocytes from senescence or apoptosis, reducing the functional cellular component of ageing tendon. Peffers *et al.* identified altered lncRNAs with age in human Achilles tendon. Of these, X (inactive)-specific transcript (XIST) was one of the most upregulated in ageing.^23^ The XIST gene is an example of a pseudogene that has been ‘resurrected’ as a lncRNA, having made the transition from protein coding to non-coding regulatory gene.^57^

#### Pseudogenes

Pseudogenes are DNA sequences closely related to actively transcribed genes, but that have typically lost their protein coding function. This is either through mutation, evolutionary processes such as duplication and divergence, or retro-transposition of mRNA from the parent protein-coding gene that is subsequently integrated back into the genome, where, lacking upstream regulatory regions, they become functionally silent.^57^ Historically, these regions of DNA were considered as remnants of redundant or failed genes and consequently, non-functional ‘junk’. However, it has now been shown that, where the appropriate upstream machinery is present, pseudogenes are actively transcribed. Because they produce mRNA in an antisense orientation, capable of hybridizing with their complimentary paralogous mRNAs, they consequently possess the ability to regulate gene expression.^57^ They show a degree of conservation between species, indicating positive selection pressure consistent with biological importance. Furthermore, they are recognized to interact with the RNA interference pathway, either through cleavage of the transcript to generate large numbers of small interfering RNAs, or by acting as miRNA decoys or sponges, preventing miRNAs from interacting with other functionally coding transcripts. Peffers *et al.*^23^ identified alteration of 12 pseudogenes in ageing human Achilles tendon. These were all functionally unannotated, but this study raises the possibility that pseudogenes are a relevant epigenetic influence in tendon ageing. It should be noted that the vast majority of pseudogenes identified as differentially expressed (DE) with ageing are unannotated and/or poorly understood in terms of function, reflecting the lack of research into these molecules and the almost complete lack of research into their tendon-specific functions.

### DNA methylation

**Fig. 2:**
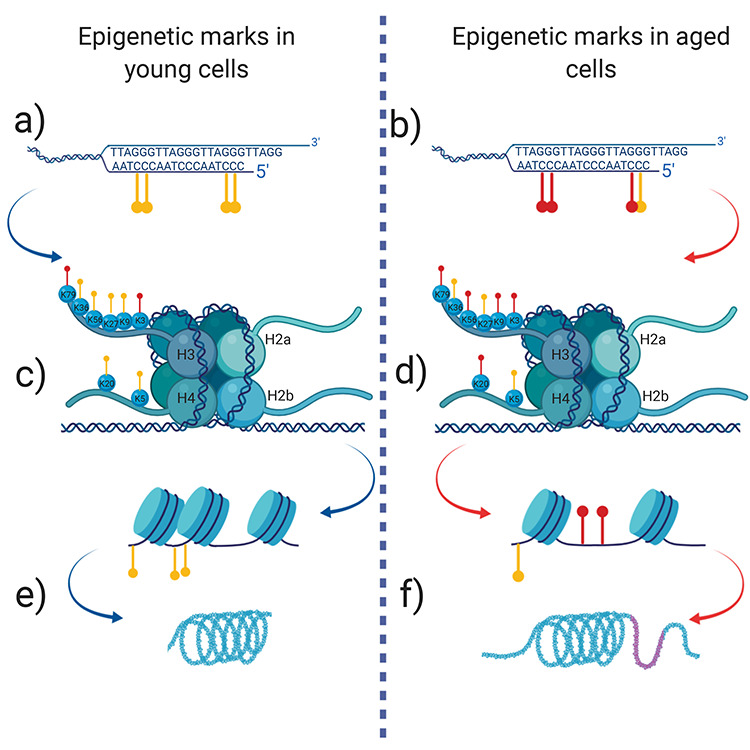
Schematic representing the aberrant DNA methylation signatures in ageing. **A** and **B** show changes in the methylome at the nucleotide level. Loss of methylation marks are seen on a global level however, hyper-methylation occurs specifically at promoter sites. **C** and **D** changes in the methylation of lysine residues on heterochromatin H3 and H4 change the conformity of the nucleosome and alter the accessibility of transcriptional factors to DNA. D. Aged cells contain a more hypomethylated histone tail. (**E** and **F**) Altered nucleosome compactness leads to abnormal chromatin formation where chromatin are not stable, leading to aberrant gene expression. (F) Demonstrates ‘aged’ chromatin where the tightly coiled chromatin (as seen in E) has lost its physiological compression. Image created with BioRender.com.

Ageing affects the DNA methylation status of nearly all cells of all organs. Tendon tissue deteriorates in a very specific manner compared to other tissues in the body, suggesting a programmed mechanism is altered due to ageing. DNA methylation can act as a form of gene expression suppression through two mechanisms; the deposition of the methyl group onto the CpGs interferes with the binding of transcription factors, or the methyl group can act as a ‘beacon’ for transcription factors, resulting in dynamic alteration of gene expression (Fig. 2). These methylation patterns and resultant effect on transcription have been hypothesized to be linked to CpG density, and display tissue type specificity. Studies have identified a tissue specific methylome. There is some conservation of methylation deposition, with 2% hypermethylated sites in 17 human tissues, 15% hypomethylated sites located proximal to transcription start sites.^58^ These tissue specific methylation patterns could explain characteristic cellular phenotypes, and their relationship to cellular function, since this directly affects the transcriptome.

DNA methylation, the addition of a methyl group (–CH_3_) to a 5′ cytosine of a CpG dinucleotide (mCpG), offers the cell epigenetic control void of mutations. For this reason DNA methylation has been associated with gene expression, with a reported 60% of human genes and 40% of tissue specific genes associated with CpG islands.^59^ However, DNA methylation does not occur at every given CpG site, rather, the ‘pattern’ of methylation alludes to a specific function. Therefore, (methylated CpG) could represent a mechanism enabling the phenotype of the cell, through selective repression and expression of transcripts in a cell cycle in a need-dependent manner. Such action is the result of the mCpG cluster blocking the binding of transcriptional apparatus or behaving as a beacon for transcriptional machinery, thus dynamically altering the expression of genes, solely dependent on where the mCpGs are located along the gene.

With the advancement of high throughput DNA technologies, terminology around CpG methylation patterns has evolved. The CpG clusters can be identified as ‘islands’, ‘shores’, ‘seas’ and ‘shelves’.^60^ CpG islands are defined as 1 kb regions of high CpG density, usually found near promoters; shores are within the 2 kb sequence neighboring the islands with seas and shelves being flanked further from shores, with occurrence of CpGs decreasing in density the further away from the island it is.^60^

The search for DNA methylation within the parameters stated in the methods yielded the papers in (Table 3). DNA methylation in healthy ageing has been previously investigated in many tissues, with results from this high throughput method of genomic interrogation producing the ageing DNA methylation clock.^61^ The majority of studies identified in this review focused on changes in diseased and healthy tissue. While there are no published studies interrogating ageing in tendon tissue and global DNA methylation, the pathological link between age-related aberrant systems in cancer, and the known similarity of dysfunctional cellular processes in ageing, could help identify the mechanism of deterioration evident in tendon ageing.

**Table 3:** Table of studies that have investigated DNA methylation in tendon tissue and cells using a targeted approach (2009–2020)

Study design	Age	Tendon type	Technique used	Key findings	Reference
12 per group; C57/Bl6 males	12 week mature	Achilles tendon	Methyl miniseq, global	Transcript modulation for 15 of the genes identified by differential promoter methylation makes it likely that the activity of the protein products of these genes was involved to some degree in the pathogenesis of tendinopathy.	^26^
Young; *n* = 4 (21.8 years ± 2.4SD), Old; *n* = 4 (65.5 years ± 8.3SD) MSCs	21.8–65.5 years	Mesenchymal stem cell	450 k Illumina methylation array	50% of the top 20 differentially methylated loci contained transcription factors, suggesting altered transcriptional regulation and ageing may be controlled through methylation events.	^7^
10 healthy, 10 patellar tendinopathy, male, Caucasian	19–41 years (age matched groups)	Patellar tendon, proximal tendon, controls were patients undergoing ACL reconstruction, PT from patellar tendinopathy	Targeted pyrosequencing	A significant difference in DNA methylation between control and PT group at the CpG site 4(+65 bp) upstream of the MMP11 first exon.	^63^
10 healthy, 10 patellar tendinopathy All male Caucasians	19–41 years age matched groups	Patellar tendon, proximal tendon, controls were patients undergoing ACL reconstruction from PT from patellar tendinopathy	Pyrosequencing Targeted	Altered methylation state seen in patellar tendinopathy group at one site upstream of ADAMTS4 (−2995 CpG).	^64^
40 patients undergoing arthroscopic rotator cuff repair; 11 patients in control group	30–70 years	Tendon from rotator cuff, Central cuff,^64^ posterior cuff (PC), and anterior cuff (AC)	Targeted pyrosequencing	Increased methylation evident in CpGs of MMP9 and MMP13 in AC samples compared to CC and PC, consistent with the dynamic expression of these genes.	^66^

ACL, anterior cruciate; PT, patellar tendon ligament; ADAMTS4, a disintegrin and metalloproteinase with thrombospondin motif 4; MMP, matrix metalloproteinase.

While DNA methylation studies specific to ageing are rare some relating to tendinopathy have been undertaken. Using direct methods to identify DNA methylation and differential gene expression in murine tendinopathy, decreased promoter methylation at six locations was revealed. Trella *et al.* identified CpG hypomethylation at CpG islands in promoter regions linked to leprel, foxf1, mmp25, igfbp6 and peg12. However, mRNA transcript expression within the same tissue revealed no significant changes in transcription for four of the five genes, suggesting the association of DNA methylation and gene expression have additional levels of regulation.^26^ One study to date, related to tendon ageing has investigated global methylome and transcriptome using an unbiased approach.^62^ In tendon constructs derived from young and old MSCs 50% of the top 20 DE CpGs were neighboring transcription factor genes, the function of which revealed the same expression profile in the cellular proteins. The primary material for the study was MSCs which themselves are poised for differentiation. Thus, perhaps the prominence of transcription factors in the results is part of the molecular architecture of the precursor material.

Three studies identified used targeted approaches to identify DE methylated CpGs associated with genes of interest in patellar and the posterior, central and anterior cuff tendons. Two of the studies used diseased and healthy patellar tendon, from healthy Caucasian male patients aged 19–41, to identify changes in the epigenome in relation to tendinopathy^63^^,^^64^ with each paper reporting a specific site; Adamts4 CpG—2995 upstream of promoter^64^ and the CpG +61 upstream of MMP11 first exon,^63^ these genes are known to translate to tendon specific proteases, involved in the maintenance of proteoglycans and the extracellular matrix. While these studies have shown that controlled analysis of the DNA sequence using a targeted approach revealed some changes in methylation at specific single CpG sites, functional significance remains to be verified, as no parallel gene expression analysis were undertaken. Tendinopathic models have long been used as proxies for aged tissue due to the similarity in the rate of degeneration of the tissue in either instance. With both conditions, ageing and injury, exhibiting decreased optimal cellular function and impaired reparatory mechanisms upon injury.^65^

The study of epigenetics on human tissue is complex due to the nature of the deposition of these marks. Age, gender, smoking status, environmental factors, hereditary conditions all play a role in the dynamic expression of all cells. Leal *et al.*^66^ 2017 also investigated these factors. Their study identified genes that were significantly altered, then the CpGs associated with these genes could be modulated. They identified differential methylation of matrix metalloproteinase 1 (MMP1) promoter and tissue inhibitor metalloproteinase 2 (TIMP2) CpG +49 downstream of the island with respect to gender, with methylation increased and decreased respectively for the genes. Further to this, smoking status was significantly correlated with increased methylation of one CpG -400 bp of island of the MMP1, and decreased methylation of CpG −19 of TIMP2 in the smoking group. This supplementary analysis further supports the need for additional information when planning such investigations to reveal the interplay of different contributory elements on the methylome, and transcriptome. While not overtly addressing the ageing phenomena epigenetic changes have been seen within tendon tissue as evidenced in the above study. Ageing tendon tissue and its reduced functionality suggests that investigation of this tissue’s epigenome can elucidate novel areas of research to underpin the mechanisms at play in ageing.

### Histone modifications

Histones are large proteins that compact DNA in a complex known as the nucleosome. Histones H2A, H2B, H3 and H4 are found in duplicate within the nucleosome and condense around 147 bp of DNA.^67^ Linker DNA can be found between each histone and a H1 histone binds to the linker DNA and histones in order to maintain the nucleosome and subsequently the overall chromatin fibre.^67^ Modifications of histones not only regulate the chromatin structure but also recruit remodeling enzymes, which utilize the energy derived from hydrolysis to reposition nucleosomes.^67^

Post-translational modification of histones allows for dynamic opening/closing of the nucleosome complex to allow/suppress transcriptional apparatus access to the DNA. Histones have been found to be expressed in different stages of the cell cycle. H2A, H2B and H3 have been found to be replication dependent and H3.3, H2A.Z cell cycle dependent. Specifically, histones H3.3 and H2A.Z are found within regulatory regions and promotor regions of genes, respectively. Histones can be methylated, acetylated, and phosphorylated.^67^ Histone methylation, unlike histone acetylation and phosphorylation, does not alter the charge of the histone at the lysine residue. Methylation, via histone lysine methyl transferases such as SUV39H1, catalyzes methylation through transfer of methyl group from S-adenosylmethionine^68^ to the lysines ε-amino group.

While the modifications themselves confer cellular control of expression, reversal of these modifications adds another layer of control. Demethylation was first identified in the lysine specific demethylase (LSD1), which required a protonated nitrogen, and Flavin adenine dinucleotide (FAD) as a co-factor. This demethylase could only de-methylate mono or di-methylated lysine residues, with further investigations it was found that combining LSD1 and co-factors like Co-REST or androgen receptor, altered the specificity and activity of the demethylase. Trimethylated histone demethylases were identified in 2006. These specific enzymes all contain a jumonji catalytic domain, utilizing Fe and α-ketohlutatate as co-factors.^69^

Investigating histone modifications in ageing tendon tissues could enable the identification of a tissue specific reduction of such methyl transferases helping us to further understand the mechanism behind the reduced proliferative capacity. One study investigated the effect of histone methyltransferases (G9a, G9a like protein, PR domain of zinc finger protein 2 (PRDM2), SUV39H1, SUV39H2, SETDB1/ESET) and their role in tenocyte differentiation.^70^ It was demonstrated that, expression of tendon-specific transcription factors such as Scleraxis, Mohawk, Egr1, Six1, Six2 were significantly decreased in G9a null tenocytes, as well as significantly reducing proliferative capacity.^70^ Scleraxis is a transcriptional activator of tenomodulin (Tnmd), a transmembrane glycoprotein critical for tenocyte proliferation and maturation.^71^ The study was conducted in a murine tenocyte model where G9a Flox/flox mice were produced and G9a was deleted using a Cre-expressing adenovirus. Reduced proliferative capacity is one of the hallmarks of ageing tissues, with many theories suggesting senescence as a key factor for this.^27^

Another study investigated stem cell differentiation into tendon cells. Retionic Acid Receptor,^6^ was identified as a mechanism of preserving the TSCs from spontaneous differentiation.^72^ Webb *et al.,* found that Scleraxis was one of the transcription factors that was able to mediate this and found arresting spontaneous differentiation could also be reversed when removing the RAR antagonist compounds. This is particularly of interest when understanding the biologically relevant role of Scleraxis (Scx) as a tendon specific differentiation transcriptional regulator. Thus, the arrested spontaneous differentiation in this study,^72^ as a result of histone modifications through the mediation of nuclear binding transcription factor Scx demonstrates the dynamic nature of these regulatory factors. Such studies further delineate the importance of understanding the native histone code in ageing tendon cells in order to identify areas in which interventions may be most suitable.

The other study returned paper was a genetic review of Friedreich Ataxia.^73^ The study demonstrated that symptoms of the disease includes an absence of tendon reflexes. Herein, histone deacetylase inhibitors were among the drugs currently used to manage symptoms, within this review. The use of histone modifying compounds currently being trialed as disease modifying drugs in other tissues, including tendon, is promising. However, no link was observed between histone modifications and tendon ageing specifically in this case. While there is little to no information on the direct biological significance of tendon ageing and histone modifications. Gene expression and subsequent cellular phenotype are directly mediated through a cell’s dynamic compactness of its histones; such observations need to be made in relation to the altered ageing tendon/tenocyte phenotype.

While there is little evidence of current research into the effect of some types of epigenetics on tendon ageing in other musculoskeletal tissues, more research has been undertaken. These studies could have potential implications to tendon ageing epigenetics. For example studies have investigated changing environmental factors on muscle cells.^74^^,^^75^ Such investigations are required in tendon ageing and disease as this could lead to novel findings to aid in the determination of how these specific epigenetic changes in ageing impact on tendon disease, especially as changes in the histone code can correlate to a change in the gene expression profile. In muscle, DNA methylation was increased in the myo-satellite cell population extracted from elderly patients.^76^ Furthermore, exercise induced histone acetylation of H3 in skeletal muscle through the removal of HDAC in the nucleus.^77^ Exercise has also been shown to increase induced Wnt/beta-catenin signaling through modification of histones H3k4me2 and H3Ac, known gene activation histones, and decreased modification of gene suppressing histones H3K9me2.^78^

## Conclusion and future perspectives

Epigenetic factors associated with normal age-related changes in healthy tendon is an under-researched area. The primary focus of many of the studies returned under our search terms was the influence of either mechanical loading or pathology on differential expression of biomolecular markers. Where age was reported, often it was a secondary variable consequent to differing case and control populations. The influence of age alone in these studies cannot, therefore, be elucidated. The studies in this review have still failed to determine the direct relationship of ageing to tendon tissue function. While some altered expression has been observed when identifying a set of tenocyte specific genes, ageing and functional implications have yet to be determined. Global non-biased exploratory studies need to be encouraged in order to interrogate tendon ageing specifically.

Given the wide inter- and intra-species variation in tendon structure and function, as well as between sex variance in tendon homeostasis,^17^ further work is required to investigate the influencers of normal ageing in tendon. This is particularly true in relation to the non-coding RNAs as this is a rapidly expanding area and one which is still poorly understood. Only when the normal situation is more fully elucidated can the interplay of ageing, mechanical loading and tendinopathy be understood in context.

Prior work on age-related changes is limited and often narrowly focused. With the advent of, and increasing accessibility to, powerful unbiased technologies, the potential to gain a far deeper and broader understanding of mechanistic processes involved in ageing has become a realistic possibility. With the increasing proportion of ageing individuals in the general population, this knowledge is vital in the promotion of healthy ageing. Many epigenetic studies to date have focused on very specific changes in the epigenome, histone modification, DNA methylation or miRNA expression, on the same tissue type but harvested from alternative sources. Emerging evidence suggests these investigations are crucial to unpicking these regulatory pathways. However, investigators should try to focus on collecting this data from the same source to ensure a robust epigenetic profile of the tissue in question. While this is not feasible in many applications where human tissue is required as source material, emerging projects should ensure investigations of such epigenetic interactions; DNAm/miRNA, miRNA/mRNA, DNAm/histone modifications can be properly characterized if the samples are the same in each ‘pairing’. In terms of investigating methylation of DNA/histones sample groups should be as close as possible depending on what is being investigates as age, gender, co-morbidities, weight, activity level, and ethnicity could all play a part in interpreting the results. When investigating the DNA methylome of healthy human ageing tendon tissue, gender played a role in masking DE epigenetic marks.^17^ In many analyses of DNA methylation studies, mixed gender groups have been employed and the gender bias potentially removed through removing the sex chromosomes. However, on a biological level this remains to be proven as the correct way to conduct this analysis, mostly due to the effects of a lifetime of sex-linked hormone-driven epigenetic changes on the methylome. Such changes may be modest but could enable a greater understanding in disease related analysis, especially in diseases, which affect one sex over another. In recent studies of the DNA methylation state of healthy and diseased human patellar tendinopathy, age and gender matched groups were employed for this reason.^63^^,^^64^

Histone modifications are deposited on the nucleosome in a need dependent manner as are DNA methylation marks, these changes enable the cell to express or repress relevant genes upon stimulation. Age alters the efficiency of many cellular processes, ultimately culminating in the functional decline of many cellular mechanisms. Deposition of histone marks is an example of such a mechanism, that shows age related decline in other musculoskeletal tissues, methylation of histones has been linked to histone compression.^79^ Compactness of the nucleosome is a physical barrier that enables the cell to control the expression of genes, with an open structure, the DNA is easily accessible to the transcriptional machinery. Loss of histone modifications that control the heterochromatin structure could result in the altered transcriptome of ageing tendon tissue as identified in.^23^

With the advent of high throughput technologies yielding evermore data, epigenetics and indeed ageing are both phenomena that can be addressed in tissues such as the tendon. While studies on tendon tissue ageing have demonstrated altered transcriptome and proteome, the next area of investigation should rigorously determine tendon ageing epignetics. This could be undertaken through investigating histone modifications of healthy aged samples, in order to deduce if conformational changes are responsible for altered function in ageing tendon, by way of the accessibility of the DNA to translational machinery through tertiary histone conformation. Alternatively, epigenetic modifications can also be investigated to identify whether alterations to the DNA or altered expression of small non-coding RNA are the mediators of internal cellular processes, through the direction of translational apparatus or inhibition of it.

## Data availability statement

No new data were generated or analyzed in support of this review.
